# Testicular tuberculosis presenting with metastatic intracranial tuberculomas only: a case report

**DOI:** 10.1186/1752-1947-5-100

**Published:** 2011-03-13

**Authors:** Godwin I Ogbole, Oku S Bassey, Clement A Okolo, Samson O Ukperi, Ayotunde O Ogunseyinde

**Affiliations:** 1 Department of Radiology, University College Hospital, Ibadan, Nigeria; 2 Department of Pathology, University College Hospital, Ibadan, Nigeria

## Abstract

**Introduction:**

Intracranial tuberculomas are a rare complication of tuberculosis occurring through hematogenous spread from an extracranial source, most often of pulmonary origin. Testicular tuberculosis with only intracranial spread is an even rarer finding and to the best of our knowledge, has not been reported in the literature. Clinical suspicion or recognition and prompt diagnosis are important because early treatment can prevent patient deterioration and lead to clinical improvement.

**Case presentation:**

We present the case of a 51-year-old African man with testicular tuberculosis and multiple intracranial tuberculomas who was initially managed for testicular cancer with intracranial metastasis. He had undergone left radical orchidectomy, but subsequently developed hemiparesis and lost consciousness. Following histopathological confirmation of the postoperative sample as chronic granulomatous infection due to tuberculosis, he sustained significant clinical improvement with antituberculous therapy, recovered fully and was discharged at two weeks post-treatment.

**Conclusion:**

The clinical presentation of intracranial tuberculomas from an extracranial source is protean, and delayed diagnosis could have devastating consequences. The need to have a high index of suspicion is important, since neuroimaging features may not be pathognomonic.

## Introduction

The incidence of tuberculosis (TB) has recently increased significantly worldwide, primarily because of the human immunodeficiency virus (HIV) pandemic. Controlling multidrug resistance with this surge is a major public health concern [1]. Tuberculosis remains the leading cause of death worldwide because of a single infectious agent, killing approximately two million people in one year [2,3]. Hematogenous spread to the central nervous system (CNS) and other organs may occur early in the course of infection, and 15% to 20% of extrapulmonary tuberculosis involves the CNS [4]. CNS involvement manifests as meningitis, cerebritis, tuberculous abscesses or tuberculomas, with incidence varying from one region to another [1]. Before the advent of modern neuroimaging modalities (computed tomography (CT) and magnetic resonance imaging (MRI)), the incidence of CNS tuberculosis in Ibadan, a southwestern Nigerian town [5], was estimated at 12.5%. Intracranial tuberculomas, however, are uncommon, accounting for about 0.2% of intracranial space-occupying lesions [6]. The radiologic features are nonspecific, however, and hence are difficult to diagnose without a proper medical history and a high index of suspicion [7]. We describe a case of a patient with testicular tuberculosis with multiple intracranial tuberculomas who was HIV-seronegative and was initially managed for testicular cancer with intracranial metastases.

## Case presentation

A 51-year-old African man was referred from a private facility with a two-month history of painless scrotal swelling and a one-week history of headache, drowsiness, incoherent speech, altered sensorium and low-grade pyrexia. He had no history of cough, breathlessness, weight loss, trauma or urethral discharge. He was known to have hypertension of two years' duration. An examination revealed marked enlargement of the left hemiscrotum, and the right hemiscrotum was also mildly enlarged. The testes were firm to hard in consistency, but there was no associated tenderness. Neurological examination revealed bilateral sixth and seventh cranial nerve palsies that were worse on the right side, impaired upward gaze and dysdiadochokinesia. His other systems were essentially normal. A clinical impression of a left testicular tumor with right-sided sympathetic orchiopathy and intracranial metastases was made. The patient's hematological and biomedical parameters were essentially normal, except for a raised erythrocyte sedimentation rate (52 mm/h). However, other complementary diagnostic tools such as serum lactic acid dehydrogenase (LCD) and β-human chorionic gonadotropin that are usually used [8] in such patients are not routinely available in our hospital.

**Figure 1:**
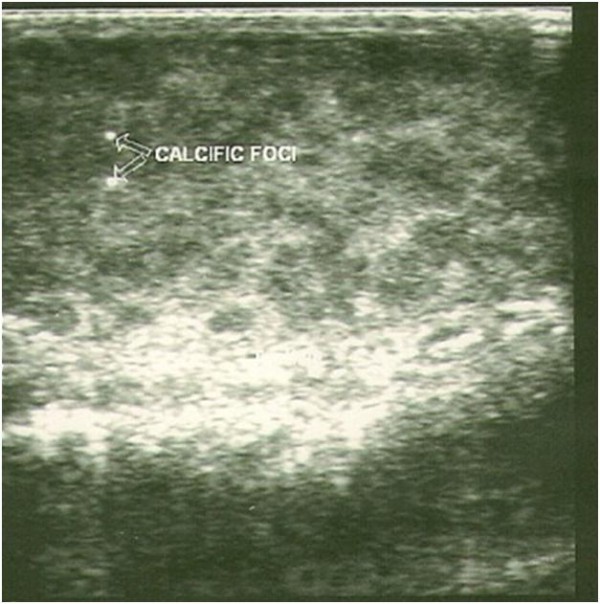
**Ultrasound image showing enlarged left testis with diffuse hypoechoic masses and multiple foci of calcification within it**.

A scrotal ultrasound showed bilaterally enlarged testes, worse on the left, with a volume of 43.5 mL and 99.7 mL on the right and left, respectively. They showed a heterogeneous echo pattern but appeared predominantly hypoechoic in nature. The left testis, in addition, showed multiple hypoechoic masses with scattered punctate calcifications (Figure 1). Doppler interrogation of both testes revealed an essentially moderate blood flow. There was no peritesticular fluid collection. An abdominal ultrasound and chest radiograph showed no abnormality. However, an abdominal CT scan, which is necessary for proper staging, was not performed because of cost constraints on the part of the patient, as our health system operates an out-of-pocket payment system. An ultrasound impression of a left testicular tumor with microlithiasis was suggested.

**Figure 2:**
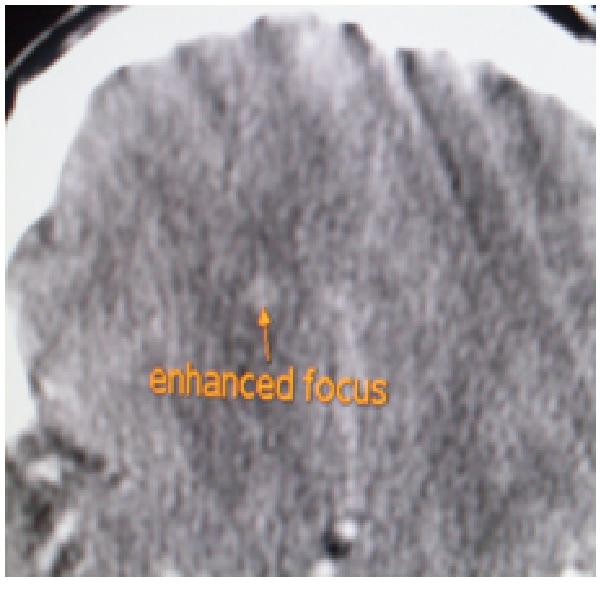
**Axial computed tomographic image showing an enhancing intracranial focus**.

Contrast-enhanced cranial CT images showed multiple widespread punctate enhancing foci, with some showing ring enhancement and minimal perilesional edema (Figure 2). The lesions involved both parietal lobes and extended to the vertex. An impression of multiple intracranial metastatic deposits, possibly from the known testicular tumor, was made.

**Figure 3:**
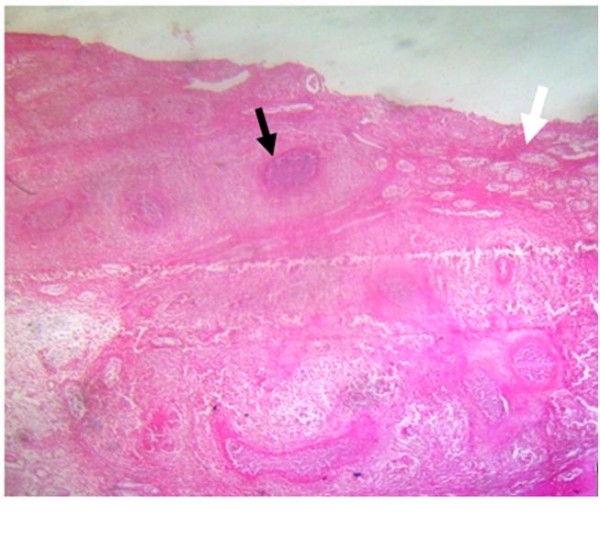
**Photomicrograph (hematoxylin and eosin stain; low-power view original magnification, ×16) of the testicular biopsy showing the testicular tissue extensively replaced by tuberculosis-induced chronic necrotizing granulomatous inflammation (black arrow) with only a few seminiferous tubules preserved (white arrow)**.

**Figure 4:**
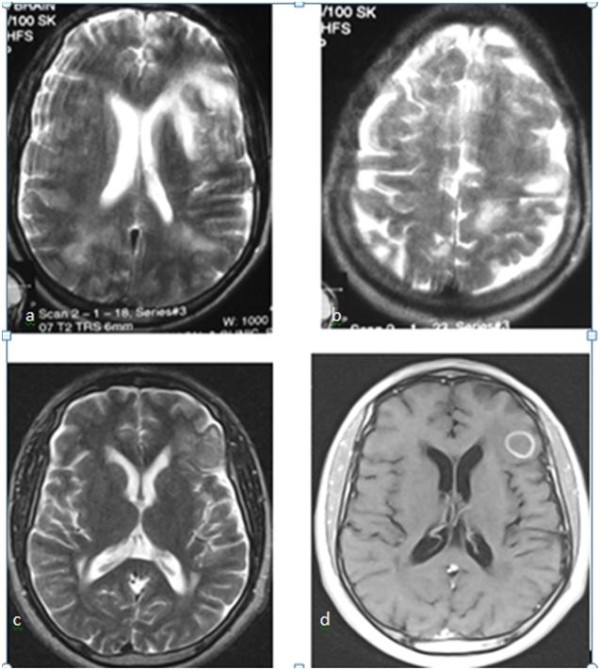
**T2-weighted axial magnetic resonance imaging (MRI) scans **(a, b) **showing extensive area of hyperintensity in the left frontoparietal region and multiple oval hyperintense lesions in both parietooccipital regions close to the vertex**. **(c, d) **T2-weighted and T1-weighted postgadolinium axial MRI scans obtained six months Post-therapy show solitary ring-enhancing tuberculoma in the left frontoparietal region with resolved edema.

The patient had undergone radical left orchidectomy and was administered intravenous ceftriaxone postoperatively. He was scheduled for radiotherapy while awaiting the histopathology report of the testicular specimen. His clinical condition nonetheless deteriorated, as he developed right-sided hemiparesis and lost consciousness on the third postoperative day. Following a histopathologic diagnosis of chronic granulomatous disease from tuberculosis (Figure 3), he was placed on antituberculous therapy (ATT), including rifampicin (600 mg/daily), isoniazid (INH) 300 mg/daily, ethambutol (1.2 g/daily) and pyrazinamide (1.5 g/daily). He also received pyridoxine (vitamin B_6_) 25 mg/daily, which is routinely given along with isoniazid. His symptoms abated, and he subsequently had sustained clinical progress with improved mental status and was discharged two weeks post-ATT for follow-up in the outpatient clinic. One week following discharge from our hospital, he developed a paradoxical response [9], with depressed level of consciousness, seizures and subsequent loss of consciousness. He was readmitted and managed with mannitol for six days as well as carbamazepine while continuing ATT. He regained consciousness and improved clinically. A cranial MRI scan obtained two weeks afterward showed a large T2 hyperintense area in the left temporal and frontal lobes with perilesional edema and multiple punctate enhancing hyperintense lesions in the periventricular regions and at the gray-white matter junction in both cerebral hemispheres consistent with tuberculomas (Figure 4a, b). He achieved significant clinical improvement on ATT and was followed up at the outpatient clinic. A six-month follow-up MRI scan showed only a solitary ring-enhancing mass in the left frontoparietal region with complete resolution of all other lesions and perilesional edema (Figure 4c). He had no complaints, and there were no observable neurological deficits.

## Discussion

Testicular tuberculosis is an unusual presentation of genitourinary tuberculosis affecting only 7% of patients with tuberculosis [10] and is usually associated with diseases in other parts of the body, such as the urinary tract, abdomen and lungs. In cases where there is no clear history of a primary disease or disseminated or other secondary diseases, testicular tuberculosis presents a diagnostic dilemma, and more often than not the correct diagnosis is made on the basis of postoperative histological samples [8]. The ultrasound features of testicular tuberculosis vary from a solitary hypoechoic mass simulating a seminoma to multiple hypoechoic masses such as nonseminomatous testicular cancer as in our patient. This diagnostic pitfall is unavoidable in the absence of other complementary diagnostic tools such as serum LCD and β-human chorionic gonadotropin which are usually raised [8]. In our patient, the puzzling features of testicular tuberculosis were compounded by the neurological symptoms of intracranial tuberculomas, which made the diagnosis of testicular tumor with intracranial metastases more likely and was readily embraced by the managing physicians. A similar line of management was reported in the literature with solitary tuberculous epididymoorchitis masquerading as a testicular tumor [8]. CNS tuberculosis has been in existence as long as tuberculosis itself. It is also endemic in Africa and other regions of the world, and recently the prevalence of tuberculosis has risen worldwide with the disease burden being compounded by HIV/acquired immunodeficiency syndrome (AIDS) cases [11].

Miyamoto *et al. *[12] reported spinal intramedullary and intracranial tuberculomas in a patient with pulmonary and testicular disease; however, to the best of our knowledge, there has been no report of testicular tuberculosis with metastatic spread to the brain alone. Since prompt diagnosis of brain tuberculomas may result in early treatment and a better prognosis for the patient, recognition of this disorder on the basis of imaging may play a critical role in patient management [13]. When brain tuberculomas are associated with meningitis, the diagnosis is more apparent and appropriate therapy can be readily instituted [14]. However, therapy may be delayed when the tuberculoma gives rise to neurological symptoms without evidence of meningitis and when the CSF profile is normal. A previous series [15] has shown that it is impossible to differentiate tuberculomas from other masses on the basis of neurological symptoms as was presented in our patient, in whom the CT images showed no evidence of meningitis. Tuberculomas may be solitary or multiple and may grow intraparenchymally, or they may have a combined meningeal and parenchymal course [1]. Since tuberculomas may be evolving, the neuroimaging appearance varies, depending on the time and stage of evolution during imaging. Tuberculomas have a central zone of caseation and necrosis surrounded by a capsule containing few bacilli [1]. Fewer than half of patients with tuberculomas have a known history of TB [1]. While some nonspecific investigations such as ESR may be positive, as in our patient, specific investigations such as acid-fast bacilli smear, CSF culture and chest x-ray may be negative, further confounding the diagnosis. However, the CSF culture may show an elevated protein level [16]. Imaging studies commonly reveal parenchymal disease involving the corticomedullary junction and periventricular regions, consistent with hematogenous spread of infection [4]. On the basis of CT, tuberculomas are peripheral, hypodense, ring-enhancing lesions sometimes showing central calcifications. Tuberculomas are usually isointense on T1-weighted images, and on T2-weighted images noncaseating lesions are bright with nodular enhancement, while caseating lesions vary from isointense to hypointense and also exhibit ring enhancement. Thus it may be difficult to differentiate tuberculomas from other intracranial lesions such as toxoplasmosis, fungal or bacterial abscesses, sarcoidosis, lymphoma or metastases from imaging features alone [17].

In our patient, the initial CT impression of metastases must have been prejudiced by an earlier ultrasound diagnosis of a testicular tumor. An intracranial tuberculoma is the least common presentation of CNS tuberculosis, and neuroimaging findings are nonspecific except where magnetic resonance spectroscopy [18] is available. Histopathological diagnosis has a prime role in early diagnosis and proper management of these patients. In this context, a detailed history and high index of suspicion are very important in directing appropriate studies, including serum LCD and human chorionic gonadotropin, necessary to diagnose this life-threatening but treatable disease [8]. The precise diagnosis in our patient was made much later on the basis of a postoperative testicular sample.

## Conclusion

The clinical presentation of CNS tuberculosis is protean, and the differential diagnosis includes other granulomatous diseases, protozoa, inflammatory disease, primary malignant lesions and metastases. Since routine tests may be negative and neuroimaging features are not pathognomonic, a high index of suspicion should be maintained in patients from regions of high prevalence presenting with extrapulmonary tuberculosis.

## Competing interests

The authors declare that they have no competing interests.

## Consent

Written informed consent was obtained from the patient for publication of this case report and accompanying images. A copy of the written consent is available for review by the Editor-in-Chief of this journal.

## Authors' contributions

GIO and OSB analyzed and interpreted the patient data regarding testicular disease and surgical findings. CAO performed the histological examination of the testicular specimen and was a major contributor in writing the manuscript. GIO and OSB reviewed the literature and wrote the first draft of the manuscript. AOO and GIO reviewed the manuscript for important intellectual content. SOU performed the sonography, provided images and made contributions to the draft. All authors read and approved the final manuscript.
